# Temporal and Spatial Patterns of Ambient Endotoxin Concentrations in Fresno, California

**DOI:** 10.1289/ehp.0901602

**Published:** 2010-05-21

**Authors:** Ira B. Tager, Frederick W. Lurmann, Thaddeus Haight, Siana Alcorn, Bryan Penfold, S. Katharine Hammond

**Affiliations:** 1 Division of Epidemiology, School of Public Health, University of California, Berkeley, California, USA; 2 Sonoma Technology, Inc., Petaluma, California, USA; 3 Division of Environmental Health Science, School of Public Health, University of California, Berkeley, California, USA

**Keywords:** bioaerosols, endotoxin

## Abstract

**Background:**

Endotoxins are found in indoor dust generated by human activity and pets, in soil, and adsorbed onto the surfaces of ambient combustion particles. Endotoxin concentrations have been associated with respiratory symptoms and the risk of atopy and asthma in children.

**Objective:**

We characterized the temporal and spatial variability of ambient endotoxin in Fresno/Clovis, California, located in California’s Central Valley, to identify correlates and potential predictors of ambient endotoxin concentrations in a cohort of children with asthma [Fresno Asthmatic Children’s Environment Study (FACES)].

**Methods:**

Between May 2001 and October 2004, daily ambient endotoxin and air pollutants were collected at the central ambient monitoring site of the California Air Resources Board in Fresno and, for shorter time periods, at 10 schools and indoors and outdoors at 84 residences in the community. Analyses were restricted to May–October, the dry months during which endotoxin concentrations are highest.

**Results:**

Daily endotoxin concentration patterns were determined mainly by meteorologic factors, particularly the degree of air stagnation. Overall concentrations were lowest in areas distant from agricultural activities. Highest concentrations were found in areas immediately downwind from agricultural/pasture land. Among three other measured air pollutants [fine particulate matter, elemental carbon (a marker of traffic in Fresno), and coarse particulate matter (PM_c_)], PM_c_ was the only pollutant correlated with endotoxin. Endotoxin, however, was the most spatially variable.

**Conclusions:**

Our data support the need to evaluate the spatial/temporal variability of endotoxin concentrations, rather than relying on a few measurements made at one location, in studies of exposure and and respiratory health effects, particularly in children with asthma and other chronic respiratory diseases.

Endotoxins are lipopolysaccharides (LPS) in the outer membranes of Gram-negative bacteria that are distributed widely on plants, in soil, water, and the intestines of humans and animals [reviewed by Myatt and Milton (2000); Spaan et al. (2008)]. Endotoxins are found in indoor dust generated by human activity and pets (Park et al. 2000, 2001b; Gereda et al. 2000a) and are also found adsorbed onto the surfaces of combustion particles (Monn and Becker 1999; Monn et al. 2003; Soukup and Becker 2001)

Inhaled endotoxins are bound by an LPS-binding protein that, in turn, binds to specific cell receptor [CD14 (Koppelman et al. 2001), a Toll-like receptor (Barton and Medzhitov 2003)], and initiates signaling pathways that lead to expression of proinflammatory cytokines (Monick and Hunninghake 2002) that result in lung inflammation, increases in epithelial permeability, and activation of systemic inflammation (O’Grady et al. 2001). Although high concentrations of aerosolized endotoxin have been recognized as a cause of lung disease in cotton (textile) workers (Castellan 1995) and swine handlers (Vogelzang et al. 1998), recent interest has focused on the complex role of nonoccupational indoor and outdoor endotoxin concentrations in the occurrence of immunoglobulin E (IgE)–mediated allergy and asthma (Koppelman et al. 2001). Biological responses to endotoxin, in theory, could lead both to suppression of IgE-mediated responses through the stimulation of interleukin 12 (Kay 1997; Verhasselt et al. 1997) and to the worsening of airway inflammation, a hallmark of asthma (Kay 1997). These effects have been reported at endotoxin concentrations lower than those found in high-risk occupational settings.

Several studies have associated elevated levels of house dust endotoxins with *a*) increased respiratory symptoms in infants (Park et al. 2001a); *b*) worsening of existing asthma that is independent of the levels of other common indoor allergens (Michel et al. 1991, 1996); *c*) decreased frequency of positive IgE-mediated skin test reactions in infants (Gereda et al. 2000b); and *d*) decreased occurrence of hay fever and positive prick skin test in children (Braun-Fahrlander et al. 2002). Rural residence, particularly on farms with animal exposure, has been reported to reduce risk of asthma (Adler et al. 2005; Douwes et al. 2007). Despite the known high levels of endotoxin in these settings (Spaan et al. 2008), definitive evidence that endotoxin, and not some other component(s) (e.g., peptidoglycans) of the microbial flora, is associated with this decreased risk has not been established (Douwes et al. 2004).

Most studies of the association between human exposure to endotoxins and allergic and respiratory disease have focused on concentrations of endotoxin in samples of house dust (Douwes et al. 2000; Gehring et al. 2001; Gereda et al. 2000a, 2000b; Michel et al. 1996; Park et al. 2001a). Few studies have evaluated the correlation between endotoxin concentrations in dust and air (Park et al. 2000, 2001b), which appears to be low—correlation < 0.3 (Park et al. 2001b).

Several recent studies have described ambient concentrations of endotoxin. Endotoxin concentrations in New Orleans, Louisiana, after flooding from Hurricane Katrina were high in flooded [3.9 EU (endotoxin units)/m^3^] and nonflooded areas (4.2 EU/m^3^) and did not differ between indoor and outdoor environments (Solomon et al. 2006). Ambient endotoxin concentrations in a large area of Southern California were below a 5.5-EU/m^3^ limit for adverse health effects in occupational settings quoted by the authors (Mueller-Anneling et al. 2004). The highest endotoxin content per milligram of PM_10_ was found in the mountain and desert areas. No seasonal patterns were detected. A 5.5-month study (August–January) at the University of North Carolina found that ambient endotoxin concentrations were greater in coarse particles [aerodynamic diameters between 2.5 and 10 μm (PM_c_)] than in particles with aerodynamic diameters < 2.5 μm (PM_2.5_). An extensive study of size-fractionated bioaerosol was performed in 20 homes in and around Palo Alto, California (Chen and Hildermann 2009). During the daytime, the highest concentrations of endotoxin were in particles with aerodynamic diameters > 10 μm (PM_10_), followed by the PM_c_ size fraction. At night, the highest concentrations occurred in the PM_c_ size fraction. Of the above studies, only the study in Southern California provides some data on spatial distributions of endotoxin based on where subjects resided; however, potential ambient sources were not investigated (Mueller-Anneling et al. 2004).

As part of a study of the effects of ambient air pollution on the natural history of children with asthma, we characterized the temporal and spatial distributions of ambient endotoxin over several years in Fresno and Clovis, California (hereafter combined as Fresno), a city surrounded by large tracts of land devoted to agriculture and animal husbandry. As part of a study to evaluate the role of ambient air pollution and bioaerosols on the natural history of childhood asthma, in this article, we focus on ambient endotoxin, its spatial distribution in relation to these sources, and the influence of meteorologic factors on daily concentrations.

## Methods

### Study location

**Figure 1:**
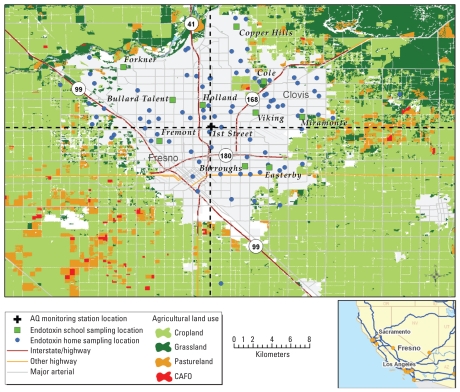
Map of study area with air quality (AQ) monitoring locations, major roadways, and agricultural land use. CAFO, concentrated animal feeding operations.

Fresno is located in the San Joaquin Valley near the southern end of the Central Valley of California. In 2006, the population was 466,700. The study area was confined to a circle with a radius of 20 km, with its center at the ambient air monitoring station operated by the California Air Resources Board (ARB) (Figure 1). The city is bound on three sides by land used primarily for agriculture and in the northeast by native vegetation. Two major interstate highways cross the study area: California State Highway 99 from northwest to southeast and Interstate 41 from north to south. The wind patterns are variable [see Supplemental Material (doi:10.1289/ehp.0901602)]. For data on collection of ambient concentration, see Supplemental Material, Figure S1 (doi:10.1289/ehp.0901602).

### Endotoxin

Daily ambient endotoxin was collected year-round at the California ARB central ambient monitoring site at 3425 First Street in Fresno (Figure 1) as part of the exposure assessment for the Fresno Asthmatic Children’s Environment Study (FACES). FACES is a cohort of 315 children 6–11 years of age at enrollment (years 2000–2005) with clinically active asthma. All subjects lived within a 20-km radius of a U.S. Environmental Protection Agency Super Site located in Fresno. Subjects were followed with biannual evaluations of respiratory health, pre- and postbronchodilator spirometry, skin prick testing (at baseline) and household surveys. Subjects also completed three 14-day panel studies (twice daily respiratory symptoms, medications, and spirometry) over three seasons based on ambient pollution concentrations in the study area. Initially, the samples were collected at the First Street site from midnight to midnight. In early 2002, the collection times were changed to 2000 to 2000 hours to coincide with the times that data were collected during panel studies and the times of the intensive sampling of 83 homes selected to cover the full range of indoor and outdoor exposures in the study community. Daily samples reported here cover 13 May 2001 through 31 October 2004.

Additional samples were collected from June 2002 to August 2003 at 10 school locations (Figure 1), with two mobile trailers outfitted by the ARB to include the instrumentation identical to that located at the First Street site. In parallel, ambient endotoxin samples were collected inside and outside 83 homes between 6 February 2002 and 22 February 2003 over 5 days (3 weekday, 2 weekend) during the 2-week panel studies of the children. Twenty-eight homes were sampled twice during two separate panels in two seasons (500 samples; mean per household = 4.3; range, 1–10). Concentrations were also measured at each location for elemental carbon (EC), PM_2.5_, and PM_10_. Concentrations of PM_c_ were determined by the difference (PM_10_ – PM_2.5_). On the residential sampling days, 24-hr samples were collected at up to eight locations: First Street, Fremont School, one other school, and up to five residences (Figure 1).

At First Street and the schools, airborne endotoxin was collected on 47-mm Teflon filters in a Partisol-Plus Model 2025 Sequential Air Sampler with a PM_10_ inlet (Rupprecht & Patashnick Co., Inc. Albany, NY). Samples were collected at a nominal flow rate of 8.33 L/min for 24 hr. At residences, 24-hr integrated samples were collected with Harvard-type PM_10_ impactors (Air Diagnostics and Engineering, Inc., Harrison, ME) at 10 L/min flow rate in a multileg sampler. One sampling leg used 37-mm Teflon filters for determination of PM_10_ mass and endotoxins. The other sampling legs employed inlets and filter media for determination of PM_2.5_ mass, sulfate and nitrate, organochlorines and EC, nicotine, metals, and polycyclic aromatic hydrocarbons. Filters were loaded and unloaded in 24-hr periods before and after the sampling period and sent to the laboratory for analysis. Collocated endotoxin data collected with the two different samplers differed by < 0.1 EU/m^3^, on average (0.09 ± 0.07 EU/m^3^).

### Analysis of endotoxin samples

Samples were analyzed using the kinetic limulus assay with resistant-parallel-line estimation method developed by Milton et al. (1992, 1997). [See Supplemental Material for details (doi:10.1289/ehp.0901602).]

Laboratory and field blanks were analyzed, and no endotoxin was detected on the 130 laboratory blanks (< 0.00001 EU/m^3^); the 165 field blanks had a mean level equivalent to a concentration of 0.01 EU/m^3^. The level of precision in 49 collocated replicates (range, 0.5–1 EU/m^3^) is comparable with that reported in other studies that used this method (Park et al. 2000, 2001a, 2001b).

### Data analysis

#### Temporal analysis

We developed a model to describe factors that influence the daily variations in endotoxin concentrations at the First Street site, based on physical variables [other than emission sources, e.g., nearby agriculture (Figure 1)] that could influence observed concentrations. Because the endotoxin concentrations were low during cooler and wetter months, data were restricted to May through October 2001–2004 (dry season) [see Supplemental Material, Figures S2–S4 (doi:10.1289/ehp.0901602)]. Three sets of variables were evaluated: *a*) wind characteristics: hourly speed and direction at specific times of day and multihour average speeds for different intervals; *b*) 24-hr average relative humidity and temperature and minimum and maximum temperature; and *c*) air recirculation, based on wind trajectory patterns during the measurement period (see Supplemental Material). Indicators for month were included to examine temporal variation in endotoxin levels over the study period. Precipitation was not considered, because there was virtually no measurable rain from June through September 2001–2004, and the monthly average precipitation was < 17 mm in May and October (see Supplemental Material, Figure S2).

We began with a linear model for the daily mean endotoxin concentrations that included the indicators for the study months (May–October) and a lag variable for the previous day’s endotoxin concentration. All subsequent models were adjusted for this lag effect to account for between-day correlation. Evaluation of autocorrelation and partial autocorrelation functions indicated that this single lag removed all autocorrelation and captured all lag associations. Residuals based on the initial model were plotted against variables from the different sets of physical variables to determine which among these was predictive of daily endotoxin concentration. Distributions of these variables were examined with respect to time and to each other. Variables were added to the model that were predictive of endotoxin concentration after adjustment for other variables included in the model, based on improvement in the log likelihood. To verify the fit of the final model, we modeled the data with the deletion/substitution/addition (DSA) model-fitting algorithm (Sinisi and van der Laan 2004). The cross-validation–based DSA is a data-adaptive, machine-learning algorithm that uses L2 loss function–based estimation to search and select models based on user-supplied parameters. The procedure mimics various forward/backward selection procedures but is more intensive in its search of the model space. The cross-validation part of its selection procedure avoids overfit models. With the same physical variables used previously, the cross-validation DSA returned a model comparable with the one obtained through our ad hoc model fit; therefore, we present results from the DSA fitting.

### Spatial analysis

We confined our analysis to 45 of 107 residential sampling days (42.7%; 46.8% of 96 days with full data across all seasons) that also had First Street endotoxin measurements and occurred during the dry season for the reasons stated previously. However, for spatial mapping of concentration patterns, we further restricted our analysis to days when six or more locations from all sites had data, which reduced the data set (*n* = 155) to 22 dry-season sampling days. The sparseness of the data at most sampling locations limits the applications of conventional spatial analysis methods; nonetheless, the data are sufficient to describe *a*) the relations between concentrations at schools and the central air monitoring station using regression equations and coefficients of divergence (CODs) [see Supplemental Material, Equation 1 (doi:10.1289/ehp.0901602)]; *b*) the range of daily spatial variability across the urban area using coefficients of spatial variations (CV) (see Supplemental Material, Equation 2); and *c*) the average spatial patterns.

Given that our study area is surrounded by areas of intensive agriculture (Figure 1), we examined associations with surrogates for potential sources of airborne endotoxin [agricultural land cover and animal-feeding operations (Spaan et al. 2006), urban parks, and schools] to assess whether proximity to these sources would have predictive power for spatial mapping. Specifically, we investigated associations of daily endotoxin levels with the area of various land covers within 10-km and 20-km radius buffers of the endotoxin measurement locations. Spatially resolved (30 × 30 m) land cover data for cropland, grasslands, and pastureland in and around Fresno (within ~ 35-km radius) were obtained from the 2001 National Land Cover Data database (Homer et al. 2004). The locations of confined animal-feeding operations (CAFOs; e.g., dairies, feedlots, and poultry facilities) were obtained from the California Department of Conservation (2006). Urban parks and schools were included because the presence of dogs and dog waste are associated with elevated endotoxin levels, and owners frequently walk dogs at these types of facilities. The locations and polygons of urban parks and schools were obtained from Tele Atlas electronic maps (Tele Atlas North America, Inc./Geographic Data Technology, Inc., Redwood City, CA). Regression analyses were conducted to assess whether proximity to these potential sources of outdoor endotoxin have predictive power for spatial mapping. These regressions did not prove useful for mapping. The endotoxin concentrations tended to be higher in the outlying areas than in the urban core; therefore, models developed from the urban core data always underestimated the higher values in the outlying areas.

We used simple spatial mapping to estimate the average spatial patterns of pollutants [see Supplemental Material (doi:10.1289/ehp.0901602)]. Spatial mapping was performed before temporal averaging because of the limited amount of temporal data in the FACES data set. Separate maps were generated on each of the 22 dry-season days. Concentration estimates were made for a grid of points with 0.25-km spacing over a 50 × 60 km domain centered on Fresno. Average dry-season maps were constructed by time averaging the daily mapped concentrations. The accuracy of the mapping method was evaluated by removing one data point at a time.

## Results

Ambient endotoxin levels showed distinct patterns with very low levels from November through March (wet season; geometric mean < 0.7 EU/m^3^) and the highest levels in June through October (dry season; geometric mean > 2 EU/m^3^) [Table 1; also see Supplemental Material, Figures S2–S4 (doi:10.1289/ehp.0901602)]. Concentrations during the dry season had a nonlinear association with both PM_c_ and PM_2.5_ concentrations; the association was stronger with PM_c_ (Supplemental Material, Figure S5); *r*^2^ = 0.44 with PM_c_ and 0.15 with PM_2.5_].

### Temporal patterns

The most important predictor of endotoxin concentrations at First Street on a given day (based on change in L2 loss function) was the endotoxin concentration on the day before (days measured from 2000 to 2000 hours and the 12-hr recirculation index (Table 2). Days with high recirculation, usually associated with summertime inversions, were associated with higher endotoxin levels. Daily endotoxin concentrations during these months also were inversely related to mean relative humidity, which is consistent with observations that the highest PM_c_ levels occur during dry atmospheric conditions.

To evaluate the applicability of the model based on First Street data, we tested the model with data from the Fremont site, the only site with a sufficient number of days of data during the dry season to evaluate the model (*n* = 120). This site is located 4.8 km southwest of the First Street monitoring site, 1.2 km east of U.S. Highway 99, and 3.6 km west of U.S. Highway 41 (Figure 1). Because Fremont had daily levels of endotoxin different from First Street, our evaluation used the variable forms from First Street (Table 2) and allowed the Fremont data to dictate the parameter estimates for each of the variables. We reasoned that if our temporal model provided a reasonable quantitative description of the physical factors that affect daily endotoxin concentrations in various parts of the study area, then the fit of the model for the Fremont site should be close to that for First Street. However, the parameters values of the model would be expected to differ, particularly the intercept term, because concentrations at Fremont were generally higher than those at the First Street site [Supplemental Material, Table S2 (doi:10.1289/ehp.0901602), dry intercept]. The data indicate that our model provides a comparable level of fit of the data at the Fremont site with that at First Street, where data were used to establish the variables and their forms: adjusted *r*^2^ = 0.52 and 0.55, respectively (Supplemental Material, Table S2). The residual plot for the Fresno model also demonstrates that this fit is much better than would have been obtained if First Street data were interpolated to the Fremont Street site (data not shown).

### Spatial distribution

Relations between daily endotoxin concentrations at First Street, Fremont, and other schools in Fresno are summarized in Table 3 and in Supplemental Material, Table S1 (doi:10.1289/ehp.0901602). Daily endotoxin levels are highly correlated (*r*^2^ ≥ 0.8) with the First Street observations at Burroughs School, but moderately correlated (0.5 < *r*^2^ < 0.8) at Copper Hills, Forkner, Fremont, Miramonte, and Viking schools, and poorly correlated (*r*^2^ < 0.5) at Bullard Talent, Cole, Easterby, and Holland schools (Figure 1). Daily concentrations at most schools are more strongly associated with levels at First Street than at Fremont (data not shown). Most of the slopes of the regression equations are < 1, indicating levels at other schools are usually lower than those at First Street (given the generally small intercepts; data not shown). However, data from the longest-operating school site (Fremont) are higher, on average, than those at First Street [(Endo)_Fremont_ = 1.03 × (Endo)_First St_ + 0.48].

Endotoxin levels at the individual homes are weakly associated with the First Street levels: *r*^2^ = 0.36 in the dry season and *r*^2^ = 0.24 in the cooler, rainy season [Supplemental Material, Table S1 (doi:10.1289/ehp.0901602)]. Home endotoxin levels are not associated as strongly with the First Street levels as those at most schools. Across the entire year, outdoor home endotoxin levels are higher than those at the First Street, on average [(Endo)_homes_ = 0.98 × (Endo)_First St_ + 0.72; *r*^2^ = 0.55], with the difference being even greater for the dry season [(Endo_homes_) = 0.77 × (Endo)_First St_ + 1.97; *r*^2^ = 0.36]. The regressions of the residential data with First Street data are not directly comparable with those for individual schools, because they included data from multiple locations.

The CODs for daily endotoxin for site pairs that involve First Street range from 0.14 to 0.40 [Supplemental Material, Table S1 (doi:10.1289/ehp.0901602)]. A similar range of CODs is evident for daily endotoxin for site pairs involving Fremont data. A COD of 0.14 corresponds to a case where one site is 33% higher, on average, than the other site; a COD of 0.40 corresponds to a case where one site is 133% higher, on average, than another site. These results indicate fairly large spatial differences in daily endotoxin concentrations between schools and the First Street site.

Table 3 lists the spatial (Pearson) correlation coefficients for daily endotoxin, PM_2.5_, PM_2.5_, EC, and PM_c_ concentrations between schools and First Street. The spatial correlation between PM_2.5_ at the First Street and PM_2.5_ at Bullard Talent, Burroughs, Easterby, Fremont, Holland, and Viking Schools is > 0.90, which indicates good correlation and the regional nature of this pollutant. PM_2.5_ and EC concentrations show higher spatial correlation than PM_c_ and endotoxin. PM_c_ and endotoxin show generally more moderate spatial correlations; endotoxin is the most spatially heterogeneous of the group.

As noted above, endotoxin has the greatest spatial variability (CVs range from 0.08 to 0.75; median = 0.27). PM_c_, with which endotoxin is most correlated, has a smaller range (0.08–0.41) and a lower median CV (0.20) [Supplemental Material, Figure S6 (doi:10.1289/ehp.0901602)]. PM_2.5_, which is a regional pollutant, has the lowest median CV (0.14) and range of daily CVs (0.05–0.39).

Regressions of daily endotoxin levels on the area of potential nearby sources showed considerable day-to-day variability and modest associations, on average. The mean *r*^2^s for associations with area of cropland, pasture land, and CAFOs within a 20-km radius of measurement locations were 0.24, 0.25, and 0.25, respectively. Association between daily pattern of endotoxin and areas of forest, grassland, schools, and urban parks were much weaker. Associations with the area of pastureland within a 10-km radius were also weaker than those for the 20-km radius buffer. Therefore, the daily spatial patterns of endotoxin are only modestly explained by cropland, pasture land, and CAFO land use. Both magnitude of endotoxin emissions from various sources and proximity of sources to the measurement site likely contribute to measured concentrations at different sites.

**Figure 2:**
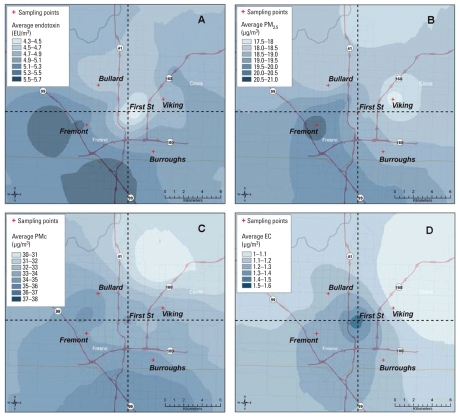
(*A*) Average endotoxin concentrations during the warm season in Fresno; (*B*) average PM_2.5_ concentrations during the warm season in Fresno; (*C*) average PM_c_ concentrations during the warm season in Fresno; (*D*) average EC concentrations during the warm season in Fresno. Colors represent different scaling for each pollutant. Bias for all pollutants ranged between 1% to 5%, on average, but measurements were less accurate for the pollutants with more spatial variability (e.g., endotoxin). The average errors in daily mapped concentrations were ± 12%, ± 20%, ± 25%, and ± 28% for PM_2.5_, PM_c_, EC, and endotoxin. The coefficients of determination (*r*^2^) were 0.86, 0.58, 0.48, and 0.34 for PM_2.5_, EC, PM_2.5–10_, and endotoxin, respectively. Dotted lines divide study area into quadrants.

The average spatial patterns based on the 22 dry-season days are presented in Figure 2A–D. The pattern for endotoxin (Figure 2A) indicates low concentrations at the First Street site (4.3 EU/m^3^) and somewhat low concentrations in the urban core north of the First Street site. The average levels are high (5.3–5.7 EU/m^3^) in the areas west and south of Fremont and Burroughs School. The high endotoxin area is mostly west of Highway 99, where the land use is primarily agricultural and includes CAFOs. Given the predominant northwesterly wind flow in the region, this pattern suggests that agricultural sources located to the west and southwest of Highway 99 generate the highest ambient levels in the region. Maps for the low and high endotoxin concentration tertiles show similar spatial patterns (not shown), whereas the map for the mid-tertile of endotoxin concentrations is fairly uniform with slightly lower levels at First Street and Fremont. When the land-use regression analysis was repeated (based on the spatially mapped 22-day warm season average endotoxin concentrations at monitored locations rather than individual daily values), we found stronger associations with the areas of CAFOs (*r*^2^ = 0.41), pastureland (*r*^2^ = 0.41), and cropland (*r*^2^ = 0.36) within 20-km radius buffers [Supplemental Material (doi:10.1289/ehp.0901602)]. These results corroborate the suspected association with agricultural land use and CAFOs.

Consistent with the correlation between endotoxin and PM_c_ concentrations, the highest concentration area for PM_c_ is found along Highway 99 and overlaps the areas of high endotoxin south of the Burroughs School (Figure 2C). A similar pattern is seen for PM_2.5_ (Figure 2B), but with a tendency toward more spatial homogeneity than for PM_c_, as noted in the COD and CV analyses. EC concentrations have the greatest spatial variability and are highest in the urban core around the First Street site and in the areas where Highways 99 and 41 intersect (Figure 2D).

## Discussion

Because of the potential importance of endotoxin in the pathogenesis of asthma (Thorne et al. 2005), we have characterized the factors that influence its temporal and spatial variability as part of a study of the natural history of asthma in children who live in an urban area surrounded by large areas of agricultural land and whose air quality is influenced by two heavily trafficked highways that pass through it.

Daily variability in endotoxin concentrations could be characterized by a common set of physical variables and variable specifications at two different locations approximately 5 km apart and at different distances from major agricultural areas that surround the study area [Figure 1; Supplemental Material, Table S2 (doi:10.1289/ehp.0901602)]. We did not have daily data on potential source emissions, which explains, in part, why our model accounted for only about 50% of each day’s variability. Furthermore, the model parameters and the form of the variables based on First Street data provided a better fit to the Fremont data than interpolation of First Street data to the Fremont site. The larger intercept for the Fremont model reflects the year-round higher levels at this location than at First Street.

Decreased relative humidity and greater recirculation of air masses with low wind speeds were associated with increased endotoxin concentrations. These conditions often coincide with summertime air inversions in the San Joaquin Valley characterized by high ozone concentrations (Blumenthal et al. 1997). It is well known that components of the bioaerosol that increase during the ozone season in Southern California can increase the occurrence of wheeze in children with asthma (Delfino et al. 1996, 1997). The specific importance of endotoxin in this setting has not yet been evaluated in any detail.

High endotoxin concentrations were measured on days with both high and low PM_2.5_ and PM_c_ concentrations. Moreover, high endotoxin concentrations (> 5 EU/m^3^) were most frequently observed on days when the concentrations of PM_2.5_ were below the current daily national standard of 35 μg/m^3^ (Figure 2B). Given the proinflammatory properties of endotoxins (Monn and Becker 1999; Shoenfelt et al. 2009; Soukup and Becker 2001), ambient endotoxin concentrations likely play a role in respiratory outcomes associated with PM. In our study area, endotoxin concentrations are highest during dry seasons. However, in other study areas with different climatic conditions and likely sources of endotoxin, the highest concentrations may be at other times of the year (e.g., Boston, Massachusetts) (Park et al. 2000). The ambient concentrations we observed are higher than airborne concentrations found indoors in other studies: Airborne endotoxin concentrations have been associated with respiratory illness in children in the first 2 years of life (Dales et al. 2006), and house dust endotoxin concentrations have been associated with wheezing (Doyle et al. 2001; Park et al. 2000, 2001a).

Endotoxin concentrations in Fresno generally are higher than those reported for 13 Southern California locations that included desert, coastal, and inland areas (Mueller-Anneling et al. 2004). Based on 8 sampling days spread over 1 year, that study reported a geometric mean concentration was 0.34 EU/m^3^ across all sites and 1.85 EU/m^3^ at the highest site (Rubidoux, California). In Fresno, the geometric mean of year-round daily samples was 1.44 EU/m^3^ at Fremont and ranged from 0.98 to 1.38 EU/m^3^ at First Street., The maximum daily concentration observed in Southern California was 5.5 EU/m^3^ (Mueller-Anneling et al. 2004) compared with 9.4, 12.4, and 16 EU/m^3^ observed at First Street, Fremont, and a Fresno residence, respectively. A June–September (1995) study in Palo Alto, California, a suburban area south of San Francisco that is not surrounded by large tracts of agricultural land, reported a geometric mean outdoor concentration of endotoxin in the PM_10_ fraction of 0.7 EU/m^3^ (Chen and Hildermann 2009), which is considerably lower than similar months over the 3 years of our study (Table 1; range of geometric means 1.95–3.77 EU/m^3^). The levels we observed are one to two orders of magnitude lower than those reported in proximity to specific sources found in our study area, such as a large dairy farms and other forms of animal husbandry (Dungan and Leytem 2009; Madsen 2006).

Our analysis shows that the spatial patterns of endotoxin, PM_2.5_, PM_c_, and EC are distinct (2A–D) and that the spatial pattern of endotoxin concentrations does not mirror any other conventionally measured pollutants in Fresno. The only similarities across pollutants are the tendency for lower concentrations in the north or northeastern areas, which are bounded by native vegetation (Figure 1) and far from Highway 99, and higher levels along and southwest of Highway 99, which is close to large areas of agriculture that include CAFOs. The differences in spatial patterns suggest differences in the locations and strength of the emission sources for different pollutants and, in the case of PM_2.5_, the influence of secondary aerosol.

The daily endotoxin values at homes and schools were not reliably predicted from measurements at First Street alone or from First Street and the four schools in the urban core. These urban core measurements (especially First Street) tend to be lower than those collected in the outlying areas (residences), which may limit their usefulness for predicting the broader pattern. Understanding of the strengths and locations of the endotoxin emission sources is quite limited; these data suggest the sources are more likely outside rather than inside the urban core. One potential major source for which we have no data relates to patterns of dog ownership and walking patterns. In indoor environments, dogs are an important source of airborne endotoxin. For example, Park et al. (2001b) reported that presence of a dog in homes accounted for 15% of the variance of airborne endotoxin, more than twice as much as any other factor. And studies have found that the presence of a dog is a major contributor to house dust and indoor air endotoxin concentrations (Campo et al. 2006; Park et al. 2001b). Although not based on a random sample of Fresno, subjects in the southwest quadrant had the lowest reported prevalence of dog ownership (11%, compared with 19%, 31%, and 31% for the southeast, northwest, and northeast quadrant subjects, respectively), which suggests that dogs are not likely to be a major source of ambient endotoxin, at least in this quadrant.

An indirect assessment of agricultural source contribution is evident in that during all dry months, endotoxin concentrations were higher at Fremont than at First Street [median (interquartile range) over study period: Fremont = 4.5 (2.6–6.0 EU/m^3^); First Street = 2.5 (1.4–4.0 EU/m^3^)]. Relative to First Street, the Fremont site is 4.1, 4.0, and 4.3 km closer to the western, southwestern, and southern boundaries of the urban core that abut on large tracts of agricultural land, respectively, with the First Street site being 6.1, 4.5, and 6 km from these boundaries, respectively. Moreover, the ratio of the quadrant-specific endotoxin concentrations relative to concentrations at First Street were greater in the southwest quadrant during both warm and dry seasons, but particularly during the dry season (data not shown). A summertime study of the dispersion of endotoxin at various downwind distances from a 10,000-cow dairy in Idaho found that endotoxin concentrations decreased exponentially and reached upwind concentrations at about 1,390 m downwind (Heinrich et al. 2003). Wind speeds were similar to those observed in our study (data not shown). Although the Fremont and First Street sites are downwind of the dominant flows, both are substantially farther downwind of sources than in the Idaho study. Thus, in our study the endotoxin concentrations would be expected to be lower than those in that study, but higher at Fremont than at First Street. However, concentrations of endotoxin measured at 10 homes closest to the west edge of the urban boundary (5.71 EU/m^3^), on average, did not have a average higher than 47 other homes further away (5.68 EU/m^3^).

Potential limitations of our spatial analysis relate to use of only 22 days in one dry season and some measurements made at different locations on different days. However, the pattern on these days is fairly robust. We found, using a leave-one-out approach, that the average spatial pattern was insensitive to the data for any specific day (data not shown). The representativeness of the spatial patterns for other time periods remains uncertain because of the lack of independent data to compare.

## Conclusion

We have demonstrated that daily ambient concentrations of endotoxin are influenced heavily by meteorology in addition to sources. Furthermore, in our study community, which is surrounded on three sides by agricultural land, endotoxin has a spatial distribution associated with proximity to CAFOs, pastureland, and cropland, and differs from PM_2.5_ (a regional pollutant) and EC (marker of traffic in our study area), but is somewhat similar to PM_c_, with which it is moderately correlated. These data support the need to evaluate the spatial and temporal variability of endotoxin concentrations, rather than relying on a few measurements made at one location in studies in which respiratory health effects associated with PM and its components are being evaluated, particularly in those studies that focus on the onset or worsening of asthma in children.

## Figures and Tables

**Table 1:** Distribution of endotoxin concentrations (EU/m^3^) by month and year.

	Geometric mean	Median	Maximum	75th Percentile	25th Percentile	Minimum
Month^a^						
January	0.28	0.30	2.59	0.57	0.15	0.05
February	0.55	0.66	4.04	1.01	0.29	0.07
March	0.69	0.83	2.64	1.12	0.37	0.12
April	0.68	0.83	6.34	1.50	0.39	0.01
May	1.17	1.26	5.00	1.89	0.89	0.03
June	1.95	1.94	7.82	2.74	1.31	0.64
July	2.49	2.43	6.77	3.53	1.74	0.75
August	2.75	3.17	9.29	4.69	1.87	0.00
September	3.77	3.87	9.43	5.35	2.68	1.17
October	2.60	3.26	8.46	4.42	1.78	0.14
November	0.59	0.64	4.03	1.01	0.32	0.09
December	0.47	0.54	3.13	0.76	0.32	0.10

Year						
2001	1.30	1.49	6.66	3.03	0.70	0.03
2002	1.38	1.72	9.43	3.60	0.60	0.07
2003	0.98	1.05	7.21	2.53	0.47	0.00
2004	1.24	1.33	8.29	2.41	0.81	0.05

a Data were accumulated between 1 May 2001 and 31 October 2004. May–October are the dry-season months; January–March and November–December are wet-season months. Precipitation is variable in April, which is not included in any season.

**Table 2:** Model^a^ for endotoxin concentrations on day t at First Street monitor.

Variable	Parameter estimate (SE, *p*)
Intercept	2.822
Endotoxin, day (t-1)^b^ (EU/m^3^)	0.6901 (0.0368, < 0.001)
Endotoxin, day (t-1)^3^ (EU/m^3^)	−0.0084 (0.0012, < 0.001)
Mean relative humidity (%), day (t)	−0.0122 (0.0058, < 0.026)
Mean relative humidity (%), day (t)^2^	−0.0010 (0.0003, < 0.001)
12-hr recirculation index^c^	1.4705 (0.4067, < 0.001)
Wind speed at 2000 hours, day (t-1)	−0.1618 (0.0330, < 0.001)
Wind speed at 2000 hours, day (t-1)^2^	−0.0211 (0.0100, < 0.023)

a Values imputed for missing days preceded and followed by a day with ambient endotoxin data and for a single day with an outlier value of 25 EU/m^3^. Number of days imputed/*n* = 20/615. *R*^2^ for model = 0.55.

b t-1 is defined as the daily endotoxin concentration over the preceding 24 hr of a given 24-hr measurement.

c Variable is the mean of six 12-hr recirculation indices: 1200 to 2400 hours day t-1; 1600 hours day t-1 to 0400 hours day t; 2000 hours day t-1 to 0800 hours day t; 2400 to 1200 hours day t; 0400 to 1600 hours day t; 0800 to 2000 hours day t. The greater the value, the greater the recirculation, and therefore, the greater the endotoxin concentration.

**Table 3:** Spatial (Pearson) correlation between daily endotoxin, PM_c_, EC, and PM_2.5_ concentrations at First Street central site and those at Fresno schools, based on full-year data.^a^

School^b^	Endotoxin	PM_c_ mass	EC	PM_2.5_ mass
Bullard Talent	0.67	0.76	0.98	0.92
Burroughs	0.89	0.95	0.94	0.99
Cole	0.66	0.57	0.97	0.89
Copper Hills	0.75	0.90	0.84	0.87
Easterby	0.52	0.88	0.66	0.93
Forkner	0.82	0.73	0.72	0.84
Fremont	0.87	0.79	0.97	0.97
Holland	0.51	0.98	0.94	0.98
Miramonte	0.75	0.81	0.90	0.84
Viking	0.71	0.92	0.92	0.99
Average	0.72	0.73	0.88	0.92

a All *p*-values in text based on two-sided test.

b See Figure 1 for sampling sites.
